# Perspectives on Healthy Eating of Adult Populations in High-Income Countries: A Qualitative Evidence Synthesis

**DOI:** 10.1007/s12529-023-10214-w

**Published:** 2023-09-05

**Authors:** Urte Klink, Victoria Härtling, Benjamin Schüz

**Affiliations:** Department of Prevention and Health Promotion, Institute for Public Health and Nursing Research, University of Bremen, Bremen, 28359 Germany

**Keywords:** Qualitative research, Systematic review, Nutrition policy, Diet, food, and nutrition, Attitude

## Abstract

**Background:**

Understanding how individuals currently perceive healthy eating is essential for developing food policies and dietary recommendations that improve the health and well-being of populations. The purpose of this qualitative evidence synthesis was to systematically outline the views and understandings of healthy eating, focusing on how foods are classified as healthy and unhealthy and what meanings are attached to food and eating by the general adult population in high-income countries.

**Methods:**

A systematic search of four electronic databases was conducted and yielded 24 relevant primary qualitative studies of generally healthy, community-dwelling adults.

**Results:**

Thematic synthesis of the included studies identified three analytic themes: constructions of healthy and unhealthy eating, considerations on dietary recommendations, and meanings attached to food and eating. Study participants generally understood what constitutes a healthy and unhealthy diet which was in line with dietary recommendations, but those of lower socioeconomic status exhibited gaps in nutrition knowledge. Participants expressed diverse opinions on dietary recommendations, including skepticism and a lack of trust. Food and eating were associated with various meanings, including pleasure, stress relief, and feelings of guilt. Moral, health, and sociocultural considerations also played a role in dietary behaviors.

**Conclusions:**

The findings suggest that improving population diet requires considering how dietary recommendations are phrased and communicated to ensure that healthy eating is associated with pleasure and immediate well-being. This review provides valuable insights for developing consumer-oriented, practicable, and acceptable food policies and dietary recommendations that effectively improve population health and well-being.

**Supplementary Information:**

The online version contains supplementary material available at 10.1007/s12529-023-10214-w.

## Introduction

There is substantial evidence for a strong link between noncommunicable diseases (NCDs) and dietary habits [1–4]. According to the Global Burden of Disease study group, suboptimal diets are a major risk factor for various NCDs, contributing to a significant proportion of global deaths and disability-adjusted life years [1]. The prevalence of overweight, obesity [5], and NCDs [6] continues to rise worldwide, including in high-income countries, despite efforts to develop and implement strategies and interventions aimed at improving population diets and health [7, 8]. Previously, it has been argued that public health nutrition interventions targeting factors on the individual level (e.g., improving nutrition knowledge through dietary guidelines) will remain ineffective if persisting environmental barriers are not addressed [9]. Therefore, it is crucial to implement effective strategies that account for the real-life experiences of individuals and address the complex interplay of factors that influence dietary behaviors.

It is widely recognized that good health can be attained and maintained by following the commonly named guidelines for healthy eating [10–12]. Determinants across the individual, social, lived, and food environments have been found to influence food choices and dietary behaviors [9, 13], and particularly social and environmental factors have been reported as barriers to healthy eating [9]. In addition to these environmental determinants, intrapersonal constructs such as beliefs, attitudes, motivations, meanings attached to foods, values, cultural and social norms, self-identity [13], and nutrition knowledge [13, 14] have also been identified to influence food-related behaviors. For instance, viewing oneself as a healthy eater was a significant predictor for healthy eating behaviors [15, 16], while attitudes toward healthy food-related behaviors and the perceived influence of diet on health were also strong predictors of healthy eating behaviors [17].

However, many of these constructs are dependent on individual perceptions of what constitutes a healthy diet [10]. Quantitative research has provided important observations into laypeople’s conceptualization of a healthy diet. In a study of Japanese adults, maintaining a balanced diet, eating plenty of vegetables, avoiding late-nigh eating, and incorporating a variety of foods were identified as key components of healthy eating [18]. Additionally, it was found that laypeople use criteria similar to experts to evaluate the healthiness of foods but tend to overlook certain factors such as saturated fat, protein, and sodium. In addition, while laypeople were able to evaluate individual food products, they struggled to assess the healthiness of entire meals [19]. Another study found that participants were divided on whether a food can be classified as healthy based on the food’s nutritional content or whether other factors affect whether a food is healthy. Factors influencing perceptions of a food’s healthiness included micronutrient content and freshness/processing [20].

A key limitation of quantitative studies in this domain is the dependence on closed questions. Qualitative research can provide more and more differentiated subjective meanings, beliefs, and attitudes, as these can be highly individual [21]. Quantitative research, such as the one mentioned above, often does not capture subjective concepts of healthy eating. Here, qualitative research provides a more in-depth and flexible approach to, for instance, investigate people’s conceptualization of a healthy diet through semi-structured interviews or focus group discussions. Several primary qualitative studies have previously investigated healthy eating perception and the meanings people attach to food and eating (e.g., [22–26]). Furthermore, evidence derived from qualitative research can be valuable in informing quantitative approaches (and vice versa), such as interventions or the assessment of common themes in large study samples.

Qualitative evidence synthesis (QES) is an umbrella term for methodologies used in the systematic review of primary qualitative research and aims to integrate findings that are often of nuanced or sensitive nature. QES allow researchers to gain a comprehensive and in-depth understanding of people’s experiences, views, beliefs, and priorities for a variety of issues, including health-related topics [27]. They do not allow quantifying or interpreting the sizes of associations, but can be helpful tools to provide systematic overviews over emerging fields with predominantly qualitative research. Recently, researchers have investigated factors perceived to influence healthy eating [9], healthy eating beliefs and the meaning of food in populations with low socioeconomic status [28], and healthy eating strategies for socioeconomically disadvantaged populations [29] by performing QES [30]. However, apart from one review published in 2005 investigating the perceptions of healthy eating [10] and another published in 2012 investigating healthy eating as discussed in qualitative research [11], no qualitative review has been conducted exploring how the general adult population in high-income countries perceive healthy eating and what meanings are attached to food and eating—and whether such perceptions are socially stratified. However, this in-depth understanding is crucial in the development of consumer-oriented, practicable, and acceptable food policies and dietary recommendations, which can effectively improve health and well-being of populations. Additionally, it is important to consider current attitudes to ensure that interventions and recommendations align with people’s needs and preferences [9].

Therefore, this QES aims to synthesize the literature regarding the views and understandings of healthy eating and the conceptualization of a healthy diet. In addition, it will explore which meanings are attached to healthy eating and foods will be explored. If feasible, differences in understanding by socioeconomic status will be investigated. The focus of this review is on the general adult population, as children and adolescents may have less autonomy in making their own dietary choices. Furthermore, the study focuses on individuals in high-income countries due to potential variations in cultural, socioeconomic, and environmental factors between high-income and low- or middle-income countries.

## Methods

A systematic review of qualitative literature was conducted according to a pre-specified protocol registered with the International Prospective Register of Systematic Reviews (PROSPERO: CRD42021269656). Thematic synthesis, as described by Thomas and Harden [31], was employed, and reporting was guided by the ENTREQ statement [32]. Ethical approval was not required as this was an evidence synthesis of existing research.

### Search Strategy

A first search was conducted in September 2021 across four electronic databases (MEDLINE, CINAHL, PsycINFO, Scopus). The search terms and their combinations were guided by the SPIDER tool for QES [33]. Terms encompassed five key search concepts: “adults,” “high-income countries,” “healthy eating,” “understanding,” and “qualitative research.” These concepts are related to the sample, phenomenon of interest, study design, evaluation, and type of research. Following a previously described approach to identifying high-income countries [9], the literature search was restricted to countries that are classified as high-income by the World Bank [34] and are also members of the Organization for Economic Co-operation and Development (OECD) [35]. The literature search was repeated in January 2023 to include recently published articles. Table S1 in the Electronic Supplementary Material provides a list of search terms.

### Eligibility Criteria

Articles were eligible if they focused on community-dwelling adults (18 years or older) as it was assumed that they can make and report on their dietary behaviors independently, and dietary recommendations are generally addressed at this group [12]. Populations composed exclusively of children, adolescents, older adults (60 years or older), migrant, and native or indigenous groups, participants with pre-existing medical conditions, or women who are pregnant or breastfeeding were excluded as these groups were assumed to conceptualize healthy eating differently and would ultimately deserve their own review. Only the layperson’s perspective of healthy eating was of interest for this review; thus, studies examining the opinions of nutrition and health experts were excluded. Studies were excluded if the focus was on participants’ views on the dietary behaviors of others (e.g., parents expressing their views on child nutrition) and perspectives on healthy eating after an intervention. Studies were only included if the objectives or outcomes explored participants’ views and understandings of a healthy diet or their relationship with food and eating and were excluded if the outcomes only indirectly related to their opinions (e.g., external factors influencing dietary behaviors, healthy lifestyles in general, weight maintenance, behavior change) or exclusively focused on the consumption of specific foods, food groups, or diets. Qualitative primary research using methods such as interviews, focus groups, open-ended surveys, and only the qualitative components of mixed methods studies were included in this investigation. Studies were required to have incorporated participant comments or quotes to allow for independent interpretation of their perspectives. Quantitative research was excluded. Non-English full-text publications were excluded from this qualitative review.

### Study Selection Process

**Fig. 1:**
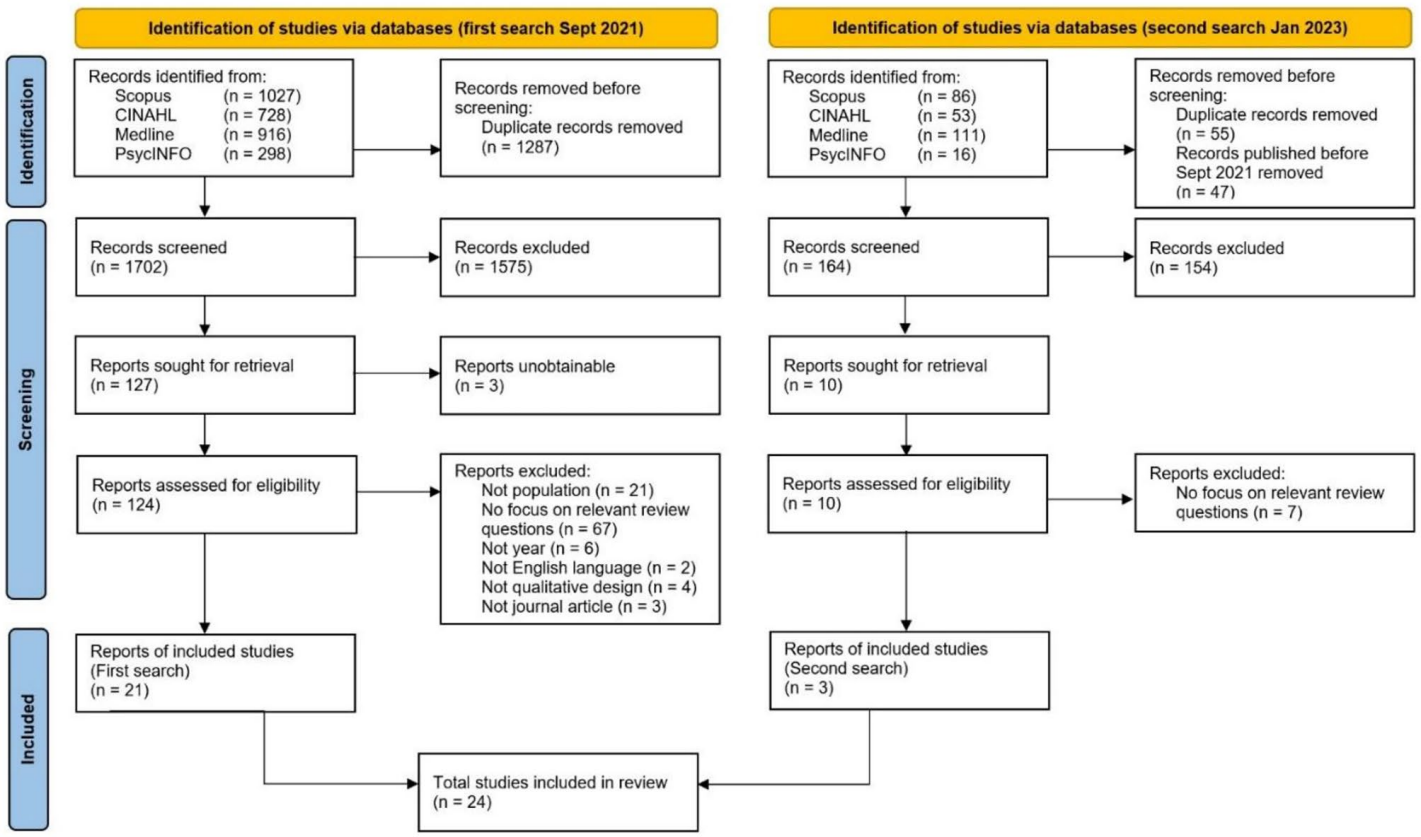
Modified PRISMA 2020 flow chart [36]

References identified through the search strategy were uploaded into EndNote version X7 (Thomson Reuters), and duplicate citations were removed by the lead author (UK). In the second search, previously identified publications were retrieved due to no date limit set in some databases and were subsequently removed. The remaining records were uploaded to the Rayyan web application for screening. Two reviewers (UK, VH) independently screened all titles and abstracts retrieved from the literature searches, excluding articles not meeting the eligibility criteria. After comparing results for the title-abstract screening, the full texts of remaining publications were independently screened by the same authors for eligibility. Publications that met the eligibility criteria were included in the qualitative synthesis. Disagreements at each stage of the selection process were resolved through discussion between the two co-authors; a third author (BS) was consulted when consensus was not achieved. Figure 1 shows the PRISMA flow diagram, which summarizes the article identification and selection process [36].

### Quality Assessment

The Critical Appraisal Skills Program (CASP) qualitative checklist was used to assess the methodological quality of included studies [37]. A qualitative checklist examines the quality across ten items, including results, methodology, research design, recruitment strategy, data collection, reflexivity, ethical considerations, and data analysis. This tool was chosen due to its widespread use in health-related systematic reviews of qualitative studies [37] and to promote consistency across reviews. Although there was an agreement not to exclude studies based on quality assessment, conducting a methodological appraisal offers transparency and insight into the strengths and limitations of the included research [30]. Two reviewers (UK, VH) independently performed the quality assessment, subsequently discussed, and any discrepancies were solved through discussion.

### Data Extraction and Synthesis

Key information from eligible studies was extracted into a Microsoft Excel template, including authors and year of publication, aim(s), study location, sample size and characteristics (gender, ethnicity, socioeconomic status), data collection methods, data analysis methods, and outcomes. Two reviewers (UK, VH) independently extracted relevant data from each study and cross-checked for accuracy.

Qualitative synthesis involves gathering and combining results from multiple primary qualitative research studies. For data analysis the thematic synthesis approach described by Thomas and Harden [31] was used. This method was selected over alternative synthesis methods (e.g., framework synthesis, meta-ethnography), as it aims to attain a higher level of analytical abstraction by rigorously investigating overlap and shared elements among studies, can be used to synthesize findings in a wide range of inquiries, and allows for the inductive and deductive identification and development of themes which collectively reflect the findings of the included studies [30]. The synthesis was conducted by following these three steps: (1) open line-by-line coding of article findings; (2) development of descriptive themes to translate concepts between studies; and (3) generation of analytical themes and interpretations to generate further ideas, explanations, and hypotheses [31].

All text under the heading “results,” “findings,” or similar was extracted from each publication and uploaded into MAXQDA Analytics Pro 2020 (Foxit Software Company) for line-by-line coding. The findings sections of five randomly selected publications were inductively coded by one reviewer (UK) to develop a code book. A second reviewer (VH) then cross-checked these codes for quality assurance. Both reviewers met to compare codes for consistency, and consensus was found through discussion. The remaining articles were coded by either UK or VH, cross-checked for accuracy by the other reviewer, and re-checked later on in the process, leading to intensive exploration of the material. New categories were added to the codebook when deemed necessary.

Codes focused on healthy and unhealthy eating, health aspects with regard to eating, dietary recommendations, meanings attached to food, and influencing factors on dietary behaviors. Differences between groups of varying socioeconomic status were checked by comparing attitudes expressed toward a discussed theme for low-income cohorts and cohorts of higher or mixed/unspecified socioeconomic status. Descriptive themes were developed by merging, grouping, and reorganizing codes and categories in an iterative process while remaining close to the original findings. Categories were organized into a thematic structure so that individual codes could be assigned to multiple descriptive themes. The lead author re-read the findings from primary studies throughout this process to ensure themes reflected data from primary studies, and all authors engaged in an ongoing discussion to refine categories and themes.

An iterative process involving inductive and deductive methods was used to develop analytical themes. The descriptive themes were analyzed in terms of their relationships to one another, and this analysis was used to address the research questions. Final descriptive themes were consolidated into mutually exclusive analytical themes.

## Results

### Summary of Included Studies

After removal of duplicates, a total of 1829 articles were identified during both literature searches, of which 24 met the eligibility criteria of this review. Detailed characteristics of included studies are presented in Table 1. Studies were published between 2007 and 2023, with more than half published within the last ten years (*n* = 13). Studies were conducted in the USA (*n* = 8), Australia (*n* = 7), Europe (*n* = 5), and Canada (*n* = 4). Study sample sizes ranged from 12 to 195 participants, totaling to 1322 participants. The populations exhibited a certain degree of diversity and included a wide range of ages (13–81 years). Of the 19 studies that had included men and women and had specified distribution, 15 studies consisted of > 50% women. While several studies (*n* = 13) did not specify the ethnic background of their population, six studies consisted of relatively ethnically diverse cohorts, and four studies solely included participants of African American or African Canadian ethnicity. Most studies (*n* = 16) provided some information on socioeconomic indicators (e.g., education, income, employment status). Four cohorts were described as low income by the respective authors. Qualitative data collection methods included interviews (*n* = 12), focus groups (*n* = 9), a combination of interviews and focus groups (*n* = 1), short written essays (*n* = 1), and online discussions on a message board (*n* = 1). Direct participant quotations included in the analysis ranged from 2 to 58 quotes per study.

**Table 1:** Characteristics of included studies and participants

Reference	Location	Sample	Population characteristics (SES; ethnicity; age)	Data collection methods	Data analysis	Research objectives
Antin and Hunt [48]	California, USA	*n* = 20 (all women)	Low income; African American ethnicity; 18–25 years old	Interviews (*n* = 20) Freelist, card sorting activity Photo-elicitation activity	Pattern level analytic technique	Factors explaining women’s food choices
Banna et al. [38]^a, b^	Hawai'i, USA (and China)	*n* = 57 US students (*n* = 55 Chinese students)	In tertiary education (undergraduate students); e*thnicity and age range not specified*	Written responses to “What does the phrase ‘a healthy diet’ mean to you?” (*n* = 57)	Directed content analysis	Meaning of healthy eating
Beagan and Chapman [56]	Nova Scotia, Canada	*n* = 38 (28 women, 10 men)	Low income: 11 of 13 participating families earning below $50,000; African Canadian ethnicity; 13–71 years old	Interviews (*n* = 38) Observation of a “typical” grocery shopping trip	Thematic analysis	Cultural food traditions Understandings of healthy and unhealthy eating, and body image
Brennan et al. [52]	Australia	*n* = 195 (119 women, 75 men, 1 non-binary)	Diverse levels of weekly disposable income, 70% currently studying; e*thnicity not specified;* 18–24 years old	Online discussion forums and conversations	Thematic analysis constant comparison approach	Young adult's beliefs and attitudes toward healthy eating
Caperchione et al. [46]^c^	Queensland, Australia	*n* = 30 (all men)	71% tertiary education, 96.7% full-time work, 73.3% household income above $100.000; e*thnicity not specified*; mean age 43.8 (SD ± 10.84)	Focus groups (*n* = 6)	Inductive analysis approach	Opinions, beliefs, and experiences regarding challenges and motivations to physical activity and healthy eating
Delaney and McCarthy [41]	Ireland	*n* = 50 (25 women, 25 men)	32% tertiary education, 34% part-time work, 22% full-time work, 30% retired; e*thnicity not specified;* 50–69 years old	Interviews (*n* = 50)	Thematic analysis	Moral space of food for older adults Understanding of the multiple meanings of food
Dong et al. [57]	Rhode Island, USA	*n* = 22 (7 women, 15 men)	*SES not specified,* adults on probation, 72.7% on food assistance; ethnically diverse; 22–64 years old	Interviews (*n* = 22)	Thematic analysis	Food access and dietary intake Knowledge about diet-health relationship Perception about healthy eating
Fergus et al. [58]	Louisiana, USA	*n* = 29 (24 women, 4 men)	Low income, 36% less than high school, 50% high school, 39% on food assistance; ethnically diverse; 18–67 years old	Focus groups (*n* = 3)	Grounded theory and cross-case analysis	Attitudes, subjective norms, perceived behavioral control, beliefs and intentions (TPB) regarding healthy eating
Kombanda et al. [43]	Australia	*n* = 38 (29 women, 8 men, 1 prefer not to say)	67% currently studying, 33% not studying, 34% full-time work, 29% part-time work; e*thnicity not specified*; 18–30 years old	Interviews (*n* = 38)	Thematic template analysis	Classification of healthy and unhealthy foods Differences in living arrangement
Koteyko [39]^d^	England, UK	*n* = 34 (20 women, 14 men)	Varying SES^g^; *ethnicity not specified*; 19–61 years old	Focus groups (*n* = 5)	Discursive analysis, using concepts of interpretative repertoire, subject position	Perspectives on healthy eating and probiotics/functional foods
Landry et al. [44]	Quebec, Canada	*n* = 92 (48 women, 44 men)	92.4% tertiary education, 41,3% family income above Can$50.000, 47% currently studying, 49% employed; 84% White ethnicity; 19–49 years old	Focus groups (*n* = 12)	Thematic content analysis	Definitions of eating pleasure and healthy eating
Lee et al. [62]	Australia	*n* = 50 (36 women, 14 men)	*SES and ethnicity not specified*; 18–72 years old	Focus groups (*n* = 9)	Thematic template analysis	Relationship between food and mood Meanings assigned to food
Lucan et al. [47]	Pennsylvania, USA	*n* = 33 (18 women, 15 men)	Low income, 62% high school or less, 58% household income below $25.000, 66% employed; African American ethnicity; 18–81 years old	Interviews (*n* = 33)	Thematic analysis	Definitions of healthy eating Strategies to improve eating behaviors
McKenzie and Watts [60]	Scotland, UK	*n* = 31 (23 women, 8 men)	52% tertiary education, 61.3% income level below £40,000; *ethnicity not specified*; 18–77 years old	Interviews and food diaries (n = 31)	Thematic analysis	Relationship between food ideals, food rules and strategies to maintain healthy eating patterns
Mete et al. [42]	Australian Capital Territory, Australia	*n* = 23 (19 women, 4 men)	69% tertiary education; e*thnicity not specified*; 25–60 years old	Interviews (n = 23)	Thematic analysis	Definitions of healthy eating How healthy food choices are translated into everyday life
Niva [45]^d^	Helsinki, Finland	*n* = 45 (25 women, 20 men)	*SES and ethnicity not specified*; 40–74 years old	Focus groups (n = 8)	Inductive and deductive analysis approach	Consumer interpretations of healthy eating and functional foods
Pettigrew et al. [50]^a^	Western Australia, Australia	*n* = 111	*SES and ethnicity not specified*; 40–79 years old	Interviews (n = 20) Focus groups (n = 12)	Inductive analysis approach	Perceptions of a healthy diet Diet-related beliefs and behaviors
Ristovski-Slijepcevic et al. [59]^e^	British Columbia and Nova Scotia, Canada	*n* = 105 (72 women, 33 men)	*SES and age range not specified*; 24.8% African Canadians, 32.4% Punjabi immigrants, 42.8% European Canadians, adults	Interviews (n = 105)	Thematic analysis	Experiences, interpretations and reasoning around healthy eating
Schoenberg et al. [61]^f^	Kentucky, USA	*n* = 99 (71 women, 28 men) (*n* = 20 key informants)	19% less than high school, 40% high school, 40% more than high school, 62% income level below $40.000; 88% White ethnicity; 18–71 + years old	Focus groups (n = 8)	*Unclear*	Perceptions of healthy eating Determinants of healthy food intake Suggestions for healthy eating programs
Sellaeg and Chapman [49]	British Columbia, Canada	*n* = 12 (all men)	Varying SES^g^; European Canadian ethnicity; 27–47 years old	Food journals and interviews (n = 12)	Constant comparative method	Interrelationships among daily food practices, food-related ideals, social context of masculinity and living alone
Sogari et al. [51]	New York, USA	*n* = 35 (23 women, 12 men)	In tertiary education (student sample); 80% White ethnicity; 18–25 years old	Focus groups (n = 6)	Thematic analysis	Barriers and enablers of healthy eating behaviors
Stephens et al. [40]	Victoria and New South Wales, Australia	*n* = 30 (all men)	50% tertiary, 50% non-tertiary education; *ethnicity not specified;*18–60 years old	Interviews (n = 30)	Qualitative description	Factors potentially contributing to educational inequalities in men's eating behaviors
Winham et al. [55]	Arizona, USA	*n* = 97 (58 women, 39 men)	13% high school or less, 57% Bachelor’s degree or higher, 53% income level below $50.000, 32% at or below poverty line; African American ethnicity; 25–60 years old	Survey on lifestyle and dietary habits (n = 97) Focus groups (n = 14)	Grounded theory	Views of food choices and use of traditional African-American foods
Wood et al. [53]	Wales, UK	*n* = 46 (all women)	Low to medium SES; *ethnicity not specified;* 41.3% ≤ 30 years old, 58.7% > 30 years old	Interviews (n = 46)	Framework analysis	Mothers' understanding of recommendations for healthy eating

*SES* socioeconomic status, *SD* standard deviation

^a^Number of women and men not specified

^b^Findings on Chinese students excluded from analysis

^c^Findings on physical activity excluded from analysis

^d^Findings on functional foods excluded from analysis

^e^Exclusion of remarks from and regarding Punjabi immigrants from analysis

^f^Exclusion of remarks from and regarding key informants from analysis

^g^No precise information on SES indicators was provided, although a certain level of diversity was noted by authors

Most studies (*n* = 18) met at least eight out of ten CASP criteria. Only six of the 24 articles met the criteria for all quality domains. Almost all 24 articles provided clear statements on aims, qualitative methodology, research design, and statement of findings; however, reflexivity was insufficient or unclear in 15 studies. The quality assessment outcomes can be found in Table S2 in the Electronic Supplementary Material.

### Results of Synthesis

Thematic synthesis of included primary studies yielded three main themes illuminating understanding and meaning of healthy eating and dietary recommendations: (1) constructions of healthy and unhealthy eating; (2) considerations on dietary recommendations and healthy eating messages; and (3) meanings attached to food and eating. A list of themes and subthemes with supporting participant quotes can be found in Table S3 in the Electronic Supplementary Material.

#### Theme 1: Constructions of Healthy and Unhealthy Eating

The first theme focuses on the construction of healthy vs. unhealthy eating, specifically considering what nutrients, food, food preparation methods, and food-related behaviors are classified as healthy and unhealthy and specific concepts of healthy eating. Table 2 provides an overview of healthy and unhealthy eating aspects mentioned. To improve readability, citations are omitted from the following two subthemes, but can be found in Table 2.

**Table 2:** Statements on healthy and unhealthy eating with regard to nutrients, food groups, food preparation and processing, and food-related behaviors

Healthy eating	Unhealthy eating
*Nutrients* Protein [38, 42, 43, 47, 51, 55, 59] Complex carbohydrates [42, 43] Fiber [38, 43, 46, 47, 49, 50, 55, 59] “Good” fats [43] Vitamins [38, 42, 43, 47, 59], vitamin C [43, 59], vitamin E [59], vitamin B [59] Minerals [38, 42, 43, 47, 50], calcium [43, 50, 59], iron [43]	*Nutrients* Sugar [38, 41–43, 56, 60] Fat [38, 41, 43, 47–49, 51, 55, 56, 60 ], saturated fats [43, 60] Cholesterol [55] Salt [43, 51, 56, 60] Additives, e.g., preservatives, pesticides, antibiotics, artificial sweeteners [43, 47, 49]
*Food groups/types of foods* Fruits and vegetables [38–47, 49–53, 55–61], including berries [45], salads [48, 56, 62], green vegetables [39, 52, 55] Grains [43, 47, 52], including porridge [39], oats [45, 55], rye [45], brown rice [43] and wholegrain products [39, 43, 53, 55, 58] Nuts [51, 52, 58] and seeds [45, 52] Protein-rich foods [38, 42, 51, 55, 58] Legumes [43, 52, 55], including beans [43, 47, 55] and lentils [55] Steak [40], lean meats [42, 43, 55], moderate amounts of meat [49, 50], poultry [43] Fish [41, 43, 47, 48, 50, 53, 55] seafood [40] Oils [45] Herbs [43] Water [51]	*Food groups/types of foods* Grains [43] “White” foods, including flour [53, 55], bread [61], pasta [43], rice [43], vegetables [55] Various types of meat [41, 43, 55, 57, 58] Eggs [47] High-fat foods [41, 45, 47, 49, 51, 55, 59, 61], animal fats [56], butter and margarine [47] Sugar-sweetened beverages [38, 47, 51, 57, 58, 61], alcohol [46], coffee [51] Fried foods [41, 47, 48, 51, 55, 56, 59, 60] Fast foods [38, 46–49, 55, 57 ] Processed foods [43, 46, 49, 51, 55, 61] Junk foods [47, 49, 61] Confectionary [41] and sweet foods [38, 51, 57], ice cream [51, 52], cookies [39, 46], cakes [46], desserts [51, 61], chocolate [43, 49, 51, 62] Potato chips [43, 46, 49] Soul foods [47, 55]
*Food preparation and processing* Homegrown [55, 59], homecooked [43, 51, 59] and homemade [42, 43, 45, 51] foods No or minimal processing [42–45, 51, 52, 56] Natural [42, 43, 45, 47, 59, 61] and whole [43, 51, 52] foods Baked [47, 61], broiled [47], grilled [43, 47, 53, 55], steamed [43, 47, 53, 55, 61], boiled [43], stir-fried [43] foods	*Food preparation and processing* Food prepared outside of home [43] Cooking foods for very long time [55] Frying [43, 47, 53, 55, 58, 61] and cooking with a lot of fat [55, 56]
*Food-related behaviors* According to official dietary recommendations [38, 39, 55, 59] According to one's energy expenditure [38] or individual nutritional needs [44, 45, 51, 59] Reducing intake of or avoiding fat [38, 39, 41–43, 45, 47, 50, 51, 55, 56, 58–60], saturated fats [43, 55], sugar/carbohydrates [38, 39, 43, 45, 47, 56, 57, 60], salt [38, 43, 45, 47, 51, 56, 58, 60], cholesterol [47], calories [47], protein [49] Five portions of fruits and vegetables a day [50, 53, 60] Balancing out different food groups [38, 39, 42–47, 51, 53] Eating a variety of foods [42–45, 47, 59] and colorful foods [43, 51, 55] Incorporating foods from all food groups in your diet [38, 43, 44, 47, 50, 59] Eating certain foods in moderation [43, 45–47] Eating healthy snacks [43, 51, 58] Eating regularly [38, 44, 45, 47, 49, 50] and having breakfast [51, 58] Respecting hunger and satiety cues [43, 44], eating when hungry [45] Being mindful of portion size [38, 43, 45, 46, 50, 51, 56, 58], food intake [39, 44] and eating speed [44] Enjoying your food [42, 44] and eating with others [44, 49] Eating in or cooking at home [49, 51] Taking vitamin supplements to ensure adequate nutrient supply [50, 59] Checking food labels [43, 60]	*Food-related behaviors* Deviating from a balanced [39] and varied [43] diet Overeating [43, 46, 51] High intake of unhealthy foods [43, 51] Eating too many carbohydrates [43, 56, 57], protein [49, 51], or fat [49, 51, 55] Eliminating certain food groups for weight loss [38] Skipping meals [49, 60], particularly breakfast [49, 51] Frequent snacking [43, 51], snacking between meals [41], irregular meals [51] Low fruit and vegetable consumption [51] Lack of fresh food groups [43] Eating out frequently [51] Low water consumption [51]

##### Construction of Healthy Eating

Healthy eating was viewed as positive and beneficial [38–41] and as contributing to well-being [38, 39, 42–45]. Participants discussed the healthiness of food and eating based on nutrients, food groups, food preparation and processing, and food-related behaviors.

Information about which nutrients study participants considered healthy was infrequently provided; however, protein, complex carbohydrates and fiber, healthy fats, and vitamins and minerals were regarded as healthy or essential.

In almost all studies, participants indicated that fruits and vegetables are a vital part of a healthy diet, virtually always mentioning them together. Other food groups, on the other hand, were mentioned less frequently, but wholegrain products, various protein sources, legumes, nuts and seeds, and fish were considered healthy. In some studies, participants expressed uncertainties regarding the healthfulness of certain food groups, which included meats [46, 47], starches [47], and dairy [47]. Apart from one study [47], dairy was neither mentioned as healthy nor unhealthy in any of the included studies. Statements were also made on the healthfulness of various types of meat, with some being considered healthier than others.

Natural foods and foods with a minimal degree of processing as well as home-cooked or homemade meals were considered healthy. Nutrient-saving cooking methods such as steaming and grilling (instead of frying) were also considered healthy.

A variety of food-related behaviors considered healthy were mentioned across included studies. These included eating a variety of (colorful) foods, avoiding or reducing the intake of fat, sugar, and salt, respecting hunger and satiety cues and being mindful of portion size, and eating according to one’s dietary needs.

##### Construction of Unhealthy Eating

While concepts of healthy eating were frequently described in the included studies, concepts of unhealthy eating were less detailed. In opposition to healthy foods, unhealthy foods were generally seen as negative [40, 41, 43, 48, 49], partly due to their adverse effects on health [48].

Nutrients considered unhealthy were sugar, fat and saturated fat, cholesterol, and salt. While protein was understood to be an important nutrient, overconsumption was considered unhealthy. Various food additives and residues were also mentioned as being unhealthy.

Foods and food groups considered unhealthy included fast foods, junk foods, and a variety of (highly) processed foods, such as confectionery, potato chips, and sugar-sweetened beverages. Additionally, different types of meat, high-fat foods, and, interestingly, “white” foods, such as white vegetables and white pasta, were considered unhealthy.

Similarly, foods that are highly processed, cooked for a long time, fried, or prepared with a lot of fat are considered unhealthy.

Regarding unhealthy eating habits, aspects such as skipping meals, frequent snacking, deviation from what is considered healthy, and overeating were mentioned.

##### Concepts of Healthy Eating

Balance and moderation were frequently mentioned concepts associated with the regulation of food intake.

Balance was considered a crucial aspect of healthy eating [38, 42–45, 47, 50, 51]. However, what balance means differed between studies and participants [41–44, 48, 52, 53]. According to dietary recommendations, “balance” means incorporating all food groups in differing proportions and providing the body with all nutrients required for optimal health [54]. While some studies reported that “balance” was indeed understood as eating meals that included foods from all food groups [38, 42, 43, 50], others believed that “balance” means one could compensate for unhealthy eating with healthy eating or other health behaviors later on [41, 44, 48, 52, 53].

Moderation was named as another relevant concept of healthy eating [39, 43–47, 49, 50]. Generally, foods that were perceived to be less healthy should be eaten “in moderation” [43, 44, 46, 49, 55]. Participants in several studies appeared to use this concept to control their intake of foods they considered unhealthy [41, 43, 44, 49, 50, 55] but also to justify the consumption of these foods [41, 43, 44, 50, 55].

#### Theme 2: Considerations on Dietary Recommendations and Healthy Eating Messages

The second theme focuses on study participants’ knowledge and awareness of healthy eating messages as well as their diverse attitudes and opinions regarding dietary recommendations.

##### Knowledge and Awareness of Healthy Eating Messages

Although none of the studies had formally tested participants’ nutritional knowledge, it appears that most study participants had at least a rough idea about what constitutes a healthy diet according to dietary recommendations [38, 40–47, 49–51, 53, 55–59], with some demonstrating more comprehensive nutrition knowledge [42, 43, 59]. Participants were aware of dietary guidelines [38, 39, 42, 45, 47, 50, 53, 55–57, 59, 60] and identified key messages [38, 39, 45–47, 50, 53, 55–57, 59, 60], such as eating five servings of fruits and vegetables a day [50, 53, 60] and choosing high-fiber foods [51, 59, 60]. Mentions of specific messages included in dietary recommendations can be found in Table S4 in the Supplementary Material.

Some authors explicitly mentioned gaps in nutrition knowledge in their cohorts. Participants reported inaccurate or superficial nutrition knowledge [40, 46, 47, 51, 53, 57, 58] or were unsure or did not know what foods are healthy besides fruits and vegetables [53, 58]. Particularly low-income [47, 53, 57, 58] and non-tertiary educated [40] participants exhibited a lack of nutrition knowledge, but this was not reported for all low-income participants [48, 55, 56, 61] and not for any of the cohorts or participants with higher socioeconomic status indicators [38–44, 49, 52, 55, 60], except for a student population [51] and a cohort of men [46]. Indeed, participants with higher formal education were described as having better nutrition knowledge [40, 59].

Participants reported knowing about healthy eating from a variety of sources, including relatives and friends [39, 42, 43, 48, 51, 58, 59], health professionals [55, 58, 59], school health education [43, 58, 59], food labels [43, 60], different media outlets [39, 42, 46, 53, 56, 59, 60], and the Internet [42, 52, 58, 59]. The reliability of information on nutrition from social contacts [42, 43] and various media outlets [42, 46, 58] was questioned as messages can be contradictory. Participants sought out multiple sources and drew from personal experiences to determine what dietary practices most benefitted their individual needs, while also considering cultural food traditions [59].

##### Attitudes and Opinions Toward Healthy Eating Messages

Participants held diverse views on dietary recommendations, with some expressing a lack of trust, confusion, skepticism, rejection, and resistance to these messages [39, 41, 42, 45, 46, 52, 53, 56, 59, 60, 62].

Participants acknowledged that effort, discipline, and a degree of self-control are required to adhere to dietary recommendations [39, 41, 44, 45, 49, 50, 59, 60] which indicate discrepancies between their requirements, actual dietary behaviors, and barriers in the environment. Furthermore, participants felt that dietary recommendations were unrealistic to achieve [52, 53], and in particular, the recommendation to eat five portions of fruits and vegetables daily, a prominent healthy eating message [12], was viewed as unrealistic [53].

Participants showed a lack of trust, confusion, and skepticism in government-endorsed healthy eating messages [41, 45, 52, 53, 56, 59, 60, 62]. Reasons mentioned were the lack of consideration for Black bodies [56], prioritizing personal preferences over government advice [52, 53], frequent changes in messages [46, 60], contradictory messages [41], the involvement of the meat and dairy industry in their development [59], recommendations being perceived to be based on outdated evidence [62], and putting higher value and trust in one’s physical experiences [41, 45, 59, 62].

It was reported that participants prefer to follow their own rules for healthy eating [41, 45, 56, 60, 62] according to what they perceive to be suitable for their bodies [41, 45, 56, 59, 62] or living circumstances [56, 60]. Healthiness was viewed as individually determined, leading to the conclusion that dietary recommendations are not universally applicable [45, 62], and led some to believe that since every person is different, they should not exist [62]. Scientific evidence should, however, be integrated into personal nutrition [59]. Health advice was also modified to be more applicable and acceptable [52, 53].

Instead of receiving specific instructions on what foods to favor or avoid, study participants preferred to receive specific guidance on which foods are beneficial and affordable and to gain the necessary skills to prepare them [55].

#### Theme 3: Meanings Attached to Food and Eating

This theme describes the different meanings that are attached to food and eating. A wide range of subthemes were identified, including the significance of healthy eating in daily life, eating as a pleasurable experience, food in stressful situations and as a means to provide comfort, negative feelings around food consumption, moral as well as health and well-being considerations in dietary behaviors, social significance of healthy eating, and lastly, eating as a means to construct cultural identity.

##### Significance of Healthy Eating in Daily Life

As stated above, most participants had a relatively good understanding of what constitutes a healthy diet [40–43, 45, 46, 48–53, 55–57, 59] and of the importance of healthy eating for well-being [40–43, 45, 46, 48–50, 52, 53, 62]. However, healthy eating appeared to be a daily challenge, with other aspects taking priority [41, 42, 48, 49, 51, 52, 59]. For some, healthy eating was of little importance in everyday life [52, 58].

Reviewed studies indicated that healthy eating was not always a priority or possibility [39, 40, 42, 48, 50–53, 55–59, 61], with other factors deemed more important or prohibitive [39, 40, 42, 48, 51–53, 55–59, 61], such as financial [40, 42, 46–48, 50–53, 55, 57, 58, 60, 61] and time constraints [40, 42, 46, 48–53, 55, 61], low availability of healthy foods [40, 46, 51, 53, 55–58, 61], environmental factors [40, 46, 51, 61], sociocultural meanings [41, 43, 48, 55, 56, 59], stressors [42, 51, 60, 61], various commitments [42, 48, 49, 51, 52, 61], taste and personal preferences [39–41, 44, 48, 51–53, 56, 58, 61], insufficient capacity or volition to eat according to healthy eating rules [42, 48, 49, 52, 55, 58, 62], and difficulty preparing healthy food [46, 48, 49, 51–53, 55]. Particularly low-income cohorts [48, 57, 58, 61] and a student population [51] reported insufficient financial resources for food or even food insecurity, likely resulting in poorer food choices due to higher perceived costs for healthy foods [48, 51, 57, 61].

##### Eating as a Pleasurable Experience

Eating pleasure and enjoyment of food was described as an important factor in food choice [39–41, 44, 45, 48, 51, 53, 56, 58]. Different explanations were provided as to why a particular food was viewed as pleasurable [41, 44, 45, 48, 55, 56], including a food’s cultural significance [55, 56], past experiences [41, 48], happy memories related to food [44, 48], the satisfaction they provided [48], and fulfillment of cravings [45]. Food characteristics associated with eating pleasure were taste, aesthetics, and variety [44, 45]. Food context also played a role in eating pleasure [39, 44, 48, 49], such as cooking and eating together [44, 49] or sharing a meal [44].

Some participants described the healthiness of certain foods or dishes as an added value to the pleasure they provided [44, 45]. To some, it was important to include eating pleasure when defining and promoting healthy eating [44].

Participants in other studies described the healthiness of certain foods as a factor reducing eating pleasure [39, 41, 53, 56, 58]. Participants felt that unhealthy foods tasted better than healthy foods [41, 47, 50–53, 55, 58], such as fried foods [47] and confectionary [41]. Some participants perceived healthy foods as comparatively less tasty [51, 53, 55, 58]. Adhering to healthy eating rules was perceived as a renunciation of eating pleasure [41, 53] and as an “immediate reduction in quality of life” [53].

##### Food in Stressful Situations and as a Means to Provide Comfort

In various situations, including when participants feel tired, exhausted, stressed, depressed, or experienced low mood, food was used to provide comfort [42, 48, 51, 60, 62]. Comfort-providing foods were consumed to relieve stress [48, 60, 62] or to cope with stressful situations [51, 60, 62] and to regulate mood [51, 62]. Eating was also described as a way to improve mood [62], and food was used as a reward or a treat [41, 44, 45, 50, 51, 53, 61, 62]. Participants found it difficult to adhere to healthy eating rules in stressful or emotional situations [42, 61], resulting in fewer healthy food choices [61].

There was little description of specific types of comfort foods, but some depictions hinted at foods generally understood as unhealthy, and which were also perceived as unhealthy by participants [48, 60, 62], such as chocolate [60, 62] and pizza [48]. The concept of comfort food appeared to be subjective and varied among individuals [44, 48, 62].

##### Negative Feelings Around Food Consumption

While some participants felt proud when being reminded of their efforts to eat and live healthily [41, 48, 52, 62], others described negative feelings like guilt [40, 41, 52, 62], regret [41], and shame [41, 52, 62] around food consumption. Participants experienced negative feelings due to not adhering to the healthy eating ideal that they have defined for themselves or has been defined by society [41, 52, 62] or when they were not in control [60] or lost control [39, 41, 62] of their food intake, fed children unhealthy food [40], or gained weight as a result of their perceived failings [62]. Not adhering to healthy eating rules was described as “doing something wrong,” thus inducing feelings of guilt [41].

There were also some descriptions of ways to minimize guilt, regret, and shame associated with unhealthy foods [41, 45, 52, 53, 62]. For some, these feelings could be overridden by the pleasure and enjoyment these foods provided [52]. Participants re-interpreted unhealthy foods as a physical necessity when craving these foods, thereby finding a way to justify eating unhealthier foods [45]. Consumption of negatively viewed treats was also limited to occasion and quantity [41]. When unhealthy foods are consumed, this is followed by periods of restraint and moderation, and unhealthy behaviors are balanced with healthy behaviors [41, 53], such as eating a salad after having bacon [53].

##### Moral Considerations in Dietary Behaviors

In several studies, food and eating situations were referred to as either “good” or “bad” [39–42, 45, 46, 48, 49, 52, 53, 60], reflecting moral considerations in dietary behaviors. “Good” foods, such as fruits and vegetables [48, 49] and natural foods [49], are considered healthy [39, 40, 48] and should be favored [42, 45]. Conversely, “bad” foods, such as processed foods [49] and those with high fat content [41, 49], were associated with poor health [40, 41, 48, 53] and should be avoided [41, 42, 45, 48]. Consumption of healthy food was viewed positively [39–41], while consumption of unhealthy foods [40] or overconsumption [41] was viewed negatively, leading to guilt [41, 52, 53, 60, 62] or a perception of failure [50].

Participants labeled themselves as “good” or “bad” based on their dietary decisions [39, 41, 53, 59, 60], associating being “good” with following well-known dietary guidelines [39] and exercising self-control [39, 49, 50, 59, 60]. People perceived themselves as “bad” when losing control over their eating [39], overly enjoying their food [41], or making “bad” food choices [39, 41, 52, 53], but these could later be balanced out with “good” behaviors [41, 53] or restriction [41].

Participants in several studies reported on implicit norms regarding food consumption [39–41, 52, 61]. People felt pressure to eat healthily [51, 52] or felt resented or judged by society if they made the “wrong” dietary choices [41, 52], but in certain social environments, pressure also existed to deviate from the healthy eating ideal [45, 51, 58, 60, 61].

Nonetheless, people justified consuming “bad” foods as physical necessities [45] or justifiable “transgressions from the ideal” [41]. Consumption of “bad” foods considered sinful in a cohort of older Irish adults was also normalized as a part of life [41].

##### Health and Well-Being Considerations in Dietary Behaviors

Participants recognized the impact of food consumption on health and well-being [40–43, 45, 46, 48–53, 56–59, 61, 62] and saw a connection between their dietary habits, immediate well-being, and everyday functioning [42, 43, 46, 50, 51, 57–59, 62]. Eating healthily provided a foundation to perform daily tasks [42, 50, 51, 56], while consuming unhealthy foods made one feel lethargic and tired [43, 51, 57, 58, 62]. A healthy diet was believed to reduce health problems [50, 51, 56, 57] and that an unhealthy diet was a significant factor in poor health [40, 43, 48, 50, 51, 53, 56]. Participants were also aware of the importance of diet in healthy aging [50, 52] and as a contributing factor in longevity [48, 50, 51, 58].

Participants in several studies expressed a holistic view of health and well-being by linking a combination of health behaviors to well-being [38, 45, 46, 51, 52]. Maintaining a healthy lifestyle was believed to positively affect an individual’s physical and mental well-being [38, 43, 51, 52, 59]. Healthy eating was seen as an integral part of health and well-being [38, 42, 43, 46, 52, 62], while other factors contributing to health status were exercise [38, 41, 46, 50–52], sleep [38], and smoking [52]. Consequently, participants described experiencing mental health issues when neglecting some of these health behaviors [42, 43, 62].

Participants felt that diet should be tailored to individual needs [44, 45, 62]. It was believed that each person has different physiological and psychological needs for food and nutrients [44, 45, 59, 62], and what is considered healthy for one person may differ for another [41, 45, 62]. However, it was deemed challenging to determine individual healthy eating rules [62].

Health-related benefits were identified as key incentives for participants to adopt healthy eating habits [42, 46, 50–52, 59]. People were motivated to eat healthily in order to feel better and have more energy [42, 46, 51], to maintain health and fitness levels [42, 50, 52], and to prevent or reduce the risk of diseases and/or manage health [46, 59, 61]. Participants noticed physical improvements when engaging in healthy eating, thus further motivating them to eat well [42, 46]. However, in Irish men, it was noted that health is unlikely to be a motivator as participants were optimistic about their future health [41].

Furthermore, participants made the connection that healthy eating helps maintain a healthy body weight [41, 42, 46, 48, 51, 61, 62], and this connection motivated them to eat accordingly [46, 48, 51]. Healthy eating was also equated with weight loss and dieting [61], possibly construing healthy eating as restrictive. Indeed, food restriction for weight loss was understood to negatively impact mental well-being [62].

##### Social Significance of Healthy Eating

Food and eating play a significant role in social interactions and relationships [39–41, 49, 51, 56, 58, 61]. Sharing a meal with family or friends was considered a meaningful way to stay connected and spend quality time together [56], was found to be the ideal food context [49], and an important aspect of eating pleasure [44]. Preparing a meal can also symbolize care and affection, as it requires time and effort [41, 56]. Food consumption was also linked to celebrations and joy [41].

Various social relationships were described to influence one’s eating behaviors [39, 40, 42, 48, 51, 53, 58, 61], and being around others was found to make one eat healthier [40, 49, 51, 52]. Observing others eat healthily led participants to be more mindful of their eating [51], and living with others was believed to influence one’s eating habits positively [49]. However, in some cases, participants described social situations in which eating unhealthy food was expected or encouraged [45, 51, 58, 60, 61].

As indicated by several cohorts, women traditionally hold a significant responsibility for food preparation and provision within the family unit [40, 48, 49, 53, 55, 56, 59–61], and female family members or partners were described to have a positive influence on the eating behaviors of the family [40, 49, 51, 61]. However, there were indications that this role may be changing as more men participate in food tasks [40, 49, 60] and is further underlined by the perception that healthy eating is no longer in conflict with masculinity [40]. Nonetheless, women described that they had to negotiate with family members to ensure that their notion of nutritional adequacy was followed and that healthier foods were consumed [53, 55, 58, 61].

Participants reported that childhood food experiences shaped their eating practices and food preferences as adults [41, 48, 50, 55, 60, 61]. In several cohorts, participants believed that eating habits are formed in childhood [48, 51, 55, 61, 62], and parents greatly influence their children’s eating habits [40, 42, 48, 51, 55, 58–62]. Therefore, parents should set a positive example to promote healthy eating habits in their children [40, 42, 51].

##### Eating as a Means to Construct Cultural Identity

While most included studies lacked discussion on the cultural significance of foods or eating practices, studies involving Black participants [48, 55, 56, 59] revealed distinct perspectives on healthy eating, dietary guidelines, and body image that differed from predominantly White cohorts. Black participants emphasized a “collective identity and cultural pride” [56] associated with their specific eating habits which were also used to demonstrate one’s cultural identity and heritage [59] and were interpreted as a means to resist assimilation [56].

Traditional Black cuisine was often described as Soul food [48, 55, 56, 59] and included flavorful, hot, and spicy foods [55, 56, 59]. Foods are frequently fried and are said to contain high amounts of fat [55, 56, 59]. Some of the traditional Black dishes mentioned in the studies were fried chicken [48, 55, 56], various “throw-away” meats such as ox tails and neck bones [48, 55, 56], salt pork, ham hooks, and cornbread [55]. Participants also identified traditional foods like sweet potatoes, black-eyed peas, and beans as healthy [55].

In contrast, Black participants described White cooking as flavorless and under-seasoned [56]. There was a belief that White people consume higher amounts of salad and vegetables, less fried foods, and opt for smaller portions and lighter meals [56]. Healthy eating was also viewed as the White way of eating [55, 56]. Thus, healthy eating may be perceived as something Black people do not do.

Black participants were aware of the connection between traditional dishes of the African diaspora and poor health outcomes [48, 55, 56, 59]. Foods were often described as unhealthy and fatty, with high amounts of cholesterol [48, 55, 59]. Despite this knowledge, the sociocultural significance of these foods outweighed health concerns [55, 56, 59]. Participants emphasized that their way of eating contributes to well-being by connecting to their culture and heritage [55, 56].

Black participants expressed skepticism toward dietary guidelines, perceiving them to be grounded in research on White individuals and not considering Black culture or bodies [56]. Healthy eating was often associated with White body norms and seen as incompatible with Black identity [55, 56]. However, in one cohort, some of the Black female participants aspired to be thin [48].

## Discussion

This systematic review of 24 primary qualitative research studies offers insights into the constructions of a healthy and unhealthy diet, knowledge and views on dietary recommendations, and meanings attached to food and eating by adult populations from high-income countries. To the best of our knowledge, this is the first QES on perceptions of a healthy diet of the general adult population. Collectively, participants of the included studies demonstrated a relatively good understanding of healthy eating by reproducing common components of dietary guidelines [12], but in some cohorts of lower socioeconomic status, gaps in nutrition knowledge could be observed. The content of dietary guidelines was recognized and understood, but participants held diverse opinions, with some rejecting them or mistrusting their messages. Food consumption and the act of eating were attributed with diverse meanings, with some foods being linked to pleasure, stress relief, or feelings of guilt. Participants made a connection between their dietary intake and immediate well-being and understood that their diet affects their long-term health status. In addition, social, cultural, and moral considerations also played a role in dietary behaviors. Overall, this research contributes to the field of behavioral medicine by providing comprehensive insights into individuals' perceptions of healthy eating and the factors that shape their dietary behaviors which can be beneficial for the development of food policies and dietary recommendations and can be further investigated in interventions.

Study participants had a reasonably good comprehension of what defines a healthy diet. Specifically, the regular consumption of fruits and vegetables; the concept of balance; choosing fresh, colorful, and minimally processed over highly processed foods; and avoiding foods of low nutritional quality and with high fat, sugar, and salt contents were named as important components of healthy eating. This finding is in line with guidance universally named in dietary recommendations [12] and what has previously been reported by several authors [10, 28, 63].

Overall, no major differences between groups in adults’ understanding of what constitutes a healthy diet could be identified, but gaps in nutrition knowledge were frequently reported for participants of lower socioeconomic status. It should be noted that this was not reported for all cohorts or participants of low socioeconomic status and cannot be applied to all those of low socioeconomic status. In quantitative research, however, several authors observed a positive relationship between socioeconomic indicators and nutrition knowledge [64–66], which in combination with these results highlights a substantial need for more intensive nutrition education interventions targeting low socioeconomic status groups.

Throughout the reviewed studies, there was an underlying notion that although healthy eating is essential for maintaining health and well-being, various factors often take priority or hinder individuals from following healthy eating principles on a day-to-day basis. These factors included time and money restraints, work or family commitments, social influences, sociocultural meanings attached to food, and a lack of motivation, interest, or skills to engage in healthy eating habits. While we did not observe significant differences between cohorts concerning these issues, cohorts with lower income levels, in particular, described having inadequate financial resources for food or even facing food insecurity [48, 51, 57, 58, 61], which might lead to poorer food choices due to higher perceived costs for healthy foods. It has been demonstrated that dietary behaviors are influenced by a variety of determinants, including individual, social, and environmental factors [9], and according to Teuscher et al. [67], individuals have conflicting priorities regarding healthy eating. Factors such as preserving cultural food traditions or ensuring that family members are adequately fed while facing time and money restraints may take precedence over healthy eating, even if it means consuming unhealthy foods [48, 53, 55]. Therefore, it has been emphasized that conflicting priorities, social practices, and daily circumstances need to be considered when promoting health practices [67], and interventions solely focused on individual-level factors like nutrition knowledge may not be effective in an environment that discourages behavior change [9].

Multiple studies were included that consisted of cohorts of predominantly or exclusively Black participants from the USA and Canada. Although it was not discussed in all studies with Black participants [47, 57], in several studies [55, 56, 58, 59] perspectives on healthy eating appeared to differ from those of predominantly White cohorts. It should be noted that most studies with Black participants have been conducted in individuals with low income. Therefore, results may be limited to this specific group. For Black participants, food and eating carried cultural significance, with healthy eating often being described as a White way of eating that is not embraced in Black communities [55, 56, 59]. Comparable observations have been made by one author [68] but were not discussed in other qualitative studies of similar objectives [69, 70]. Specifically, “soul food” can be viewed as a dietary practice that holds great significance for Black individuals, serving as a means of celebrating and affirming their cultural identity [68, 71]. Therefore, resisting dietary guidelines, as described in the reviewed literature [56], may be a form of resistance to cultural dominance or forced assimilation to the dominant culture [56, 72]. In light of the documented health disparities between Black and White individuals in the USA [73], healthy eating interventions should be implemented specifically for this population. It has been suggested that nutrition interventions should address commonly held beliefs regarding health and well-being and resistance to cultural dominance [56]. For interventions to be effective, they should be developed with the involvement of individuals of the target group. They must also be culturally sensitive, tailored to their lifestyles, and should focus on positive aspects of their way of eating, such as emphasizing traditional dishes from the African diaspora that are high in fiber and low in fat [55, 56, 68].

Similarly to findings by Rozin et al. [74, 75], food and eating were associated with ambivalent emotions in most study participants. On the one hand, a food’s taste and its significance in social interactions can bring about pleasure and enjoyment, while on the other hand deviating from the healthy eating ideal can cause worry, concern, and guilt.

Cooking and eating together were frequently regarded as important occasions for socializing, fostering connections, and building relationships. Sharing meals, either as a daily routine or for festivities, was typically associated with positive meanings. According to Contento and Koch [13], the social environment influences food choices and dietary behaviors. In the reviewed literature, it was suggested that being around others can have positive and negative influences on healthy eating behaviors. Indeed, research suggests that the impact of social modeling during shared meals can be beneficial or harmful to diet quality, depending on the type of modeled eating behaviors and the social context in which they occur [44, 76]. Nevertheless, sharing meals with others is linked with favorable dietary outcomes [77], while eating alone was viewed as a barrier to healthy eating [49] and is associated with lower dietary quality [78].

Participants also reported positive feelings, thoughts, and memories regarding food and eating, which led to greater enjoyment of certain foods that appear to go beyond a food's taste. Taste and eating pleasure were also found to be major factors in food choice, aligning with Contento and Koch’s model [13], which emphasizes that an individual’s biologically determined predisposition toward specific foods and past food experiences influence food choices and dietary behaviors. Preferences for certain tastes (e.g., sweet, umami) are biologically determined as a result of evolutionary processes [44, 79, 80]. Therefore, individuals tend to prefer highly palatable foods due to their high fat, sugar, and salt content, which are generally considered unhealthy [44, 81]. In the reviewed literature, participants frequently described healthy foods which are lower in fat, sugar, and salt as less tasty while preferring unhealthy foods, even though they knew of the health effects of a poor diet. This observation is consistent with a study conducted in an US American population [82], while the perception that healthy foods are tastier dominated in a French cohort [83], indicating that sociocultural factors may influence attitudes toward healthy eating. We only observed the perception that unhealthy foods taste better in studies from English-speaking countries; therefore, results may not be transferable to other high-income countries. Indeed, in the sole studies conducted in non-native English-speaking populations [44, 45], the healthfulness of food was described to contribute to eating pleasure, which underlines sociocultural differences in attitudes toward eating pleasure and healthy eating.

Participants frequently described using certain types of food, typically unhealthy ones, as a way to deal with stressful situations because they found comfort in consuming them. In stressful situations individuals were found to consume more palatable high-calorie foods than individuals in stress-free conditions [84–86]. Commonly, higher consumption of palatable foods has been attributed to their pleasurable properties which help alleviate the discomfort caused by stressful situations [87]. However, according to Pool et al. [87] stress-related eating is influenced by habits and automatic responses, described as “Pavlovian motivational bursts,” independent of hedonic pleasure or the intention to relieve stress. Therefore, exposure to an environmental stimulus associated with highly palatable food can trigger the compulsive pursuit of such foods, regardless of how enjoyable its consumption is to the individual [87]. The issue of stress-induced overeating needs to be addressed as it may promote rapid weight gain [88]. One way to reduce stress-induced or emotional eating could be to individually develop alternative strategies or habits for coping with emotional situations, as different strategies were shown to be effective [89–91]. On a macro level, people in countries with increased rates of overeating and excess weight are consistently exposed to stimuli linked to highly palatable foods, such as advertisements or fast-food restaurants [87]. It has therefore been proposed to regulate the food environment in order to reduce these stimuli [92, 93].

Moral considerations related to dietary behaviors were often reflected in the way study participants talked about food and eating situations, categorizing them as either “good” or “bad” by their perceived healthfulness. Such moral discussions around food often carry religious undertones, with concepts such as restriction being associated with “good” and indulgence being associated with “evil” [41, 52, 94, 95]. Unhealthy eating habits were often depicted as indulgent and enjoyable, while following a healthy diet was viewed as a form of sacrifice, reflecting Christian values like self-control and abstinence [41, 52]. A decline of religion’s significance in Western societies and a growing responsibility individuals face in managing their health amidst an abundance of food and rising rates of obesity have led to a shift toward “healthism” whereby individuals place great importance on health and well-being [41, 96]. Additionally, the scientifically informed discourse around healthy eating which continues to be influenced by traditional and moral codes have been described as the process of “moralization” [97, 98]. Personal food preferences and eating behavior have essentially been turned into moral decisions which was reflected in the discussions of the reviewed literature.

As a result of these moral considerations, consuming food considered unhealthy can lead to feelings of guilt, regret, or shame [41, 52, 94, 95]. Guilt, defined as a self-conscious emotion resulting from a perceived wrongdoing and often accompanied by a desire to take corrective action [99], was caused by deviating from healthy eating ideals or losing control over food intake. The association of unhealthy eating with guilt have been linked to unhealthier eating behaviors and lower levels of perceived behavioral control over eating [100, 101]. Furthermore, guilt did not appear to be a motivator for healthy eating and those who associate unhealthy food with guilt do not have more positive attitudes toward healthy eating [74, 100–102].

Based on results of a meta-analysis, it has been suggested that guilt appeal messages may be a possible strategy to promote certain health behaviors [103]. However, our findings and findings from other authors [100, 101] suggest that healthy eating messages should be cautious about inducing feelings of guilt. Instead, they should frame foods high in fat, sugar, and/or salt as occasional treats that can be enjoyed in moderation, rather than banning them altogether, and put emphasis on the pleasure of eating [100]. Indeed, describing a healthy food as both healthy and tasty was found to be a promising and effective strategy as it increased the selection of a healthy food over an unhealthy food item [104].

Growing scientific evidence has suggested the implementation of strategies that prioritize eating pleasure as a way to promote healthy eating behaviors [74, 105–108]. Further research on the effectiveness of health- vs. pleasure-oriented messages regarding healthy eating has shown that both strategies can be effective [105, 109], while Bedard et al. [108] described that conceptualizing eating pleasure as sensory experiences, mindful eating, memories, social experiences amongst others led to beneficial dietary outcomes. This suggests that strategies that go beyond the traditional health focus of dietary guidelines may be effective in improving dietary behaviors, particular in certain cultural settings.

Dietary guidelines were frequently rejected or mistrusted by participants in the included studies. As these are meant to improve population diet by educating individuals on healthy eating their effectiveness may be in question if they continue to be met by resistance. Here, consumer’s wishes for healthy eating recommendations need to be considered which may increase their credibility [110]. Based on our results, a revised approach may be necessary to improve acceptance, possibly by consistently communicating messages across media outlets and through community gatekeepers, providing strategies to implement recommendations into everyday life, taking frequently experienced barriers into consideration (e.g., financial and time constraints), and being mindful of the sociocultural significance of dietary behaviors. Using community gatekeepers as healthy eating guides can improve healthy eating habits by building trustworthiness in dietary recommendations within the community. However, more research is necessary to determine more effective strategies.

### Strengths and Limitations

The QES presented here contributes to the existing literature by offering a comprehensive overview of constructions of healthy eating, attitudes toward dietary guidelines, and the various ways meanings are attached to food and eating by the general adult population of high-income countries. This synthesis can serve as a foundation for further in-depth research on the topic of constructions of healthy eating and the significance of food. The methodical approach to thematic synthesis outlined by Thomas and Harden [4] was followed, and for reporting, the ENTREQ statement as outlined by Tong et al. [5] was adhered to. A wide range of populations in terms of geographic location, socioeconomic background, ethnicity, age, and gender were included. Most studies were published within the last decade, indicating a recent surge of interest in this topic and offering current views on healthy eating. Although the literature search initially did not include a time restriction to investigate whether there had been a change in perception over time, no relevant high-quality studies were found prior to 2007. It was evident that data saturation was reached as the publications identified through a second literature research did not offer additional insights but rather added to data richness.

This review has a few notable limitations. Qualitative reviews commonly rely on the original study author’s pre-selected participant quotes and interpretation which also presents a limitation of this review. However, to ensure this synthesis remains rooted in participant experiences, several supporting original quotes are provided for each theme. This review exclusively included populations from high-income countries which greatly limits transferability of the results to low- and middle-income countries. However, a pre-search yielded a limited number of studies conducted in these countries, therefore only studies from high-income countries were included. Most studies had a larger representation of women than men, which means that the identified themes may be more relevant and significant to women and not necessarily apply to other genders. Although the aim was to incorporate studies from diverse settings, eligible studies could only be identified with English-speaking populations except for one study from Finland [45] and another from Quebec, Canada [44]. Only reports published in English were included which may have limited the number of relevant studies included in the synthesis. Combined, these aspects limit the transferability and generalizability of the findings to other settings and countries, and results should be interpreted and used with caution. Beliefs identified in this review should not be interpreted as a comprehensive representation of how all adult populations in high-income countries perceive health eating. Instead, they should be regarded as a compilation of various attitudes toward healthy eating that can exist, with the most prominent ones highlighted in this review. We also intended to investigate for socioeconomic differences in participants’ views but due to a lack in diversity of low-income populations and a lack of clear specification of socioeconomic status in the included studies and for participants’ quotes, we were unable to do so reliably. Nevertheless, where possible, differences between groups of varying socioeconomic status were noted.

## Conclusion

This qualitative evidence synthesis on the perception of healthy eating demonstrates that while individuals are generally able to describe a healthy diet according to dietary recommendations, they attribute diverse meanings toward healthy eating and food in general, likely affecting their dietary behaviors. The results indicate that to enhance the dietary habits of a population, it is important to carefully phrase and consistently communicate dietary recommendations in a way that associates healthy eating with pleasure and immediate well-being, while also taking their daily reality into consideration. However, the current literature on perceptions and meanings of healthy eating remains limited, particularly for underserved populations. Therefore, further in-depth research is needed to gain a better understanding of perceptions and constructions of healthy eating, including specific food-related values held by different populations and their influence on diet-related behaviors. As a first step, this review provides valuable insights for developing consumer-oriented, practicable, and acceptable food policies, behavioral medicine interventions, and dietary recommendations that can effectively improve population health and well-being.

## Supplementary Information

Below is the link to the electronic supplementary material.

Supplementary file1 (DOCX 80 KB)
