# Supplementary material for: Perspectives on Healthy Eating of Adult Populations in High-Income Countries: A Qualitative Evidence Synthesis
Source: Int J Behav Med. 2023 Sep 5;31(6):923–43. doi: 10.1007/s12529-023-10214-w (PMC11588813; doi:10.1007/s12529-023-10214-w)
Supplement: Supplementary file 1 — Supplementary file1 (DOCX 80 KB) [file 12529_2023_10214_MOESM1_ESM.docx]

**Perspectives on healthy eating of adult populations in high-income countries: A qualitative evidence synthesis**

**Electronic Supplementary Material**

Table S1: Medline search strategy (Sept 14, 2021) SPIDER search terms

|  | **RESEARCH QUESTION AND RATIONALE** | **#** | **SEARCH TERMS** |
| --- | --- | --- | --- |
| **SAMPLE** | Adult populations in high-income countries according to World Bank definition [1] and OECD member states [2] | **1** | "Developed countr*" OR "Developed nation*" OR "High income*" OR "high socioeconomic" OR Australia OR Austria OR Belgium OR Canada OR Chile OR "Czech Republic" OR Denmark OR Estonia OR Finland OR France OR Germany OR Greece OR Hungary OR Iceland OR Ireland OR Israel OR Italy OR Japan OR Korea OR Latvia OR Lithuania OR Luxembourg OR Netherlands OR "New Zealand" OR Norway OR Poland OR Portugal OR "Slovak Republic" OR Slovakia OR Slovenia OR Spain OR Sweden OR Switzerland OR "United States" OR USA OR America OR England OR Scotland OR Wales OR "United Kingdom" OR UK OR "European Union" OR EU |
|  | Additional for any adult population (18+) | **2** | adult* |
| **PHENOMENON OF INTEREST** | Understanding, interpretation, and perception of healthy eating as well as meanings attached to healthy eating  Key terms/synonyms for healthy eating, diet, food | **3** | healthy AND (food OR eating OR diet)) |
| **DESIGN** | Commonly used research methods used in qualitative studies | **4** | qualitative OR interview* OR "focus group*" OR "thematic analys*" OR "content analys*" OR "mixed methods” |
| **EVALUATION** | Synonyms for outcomes: Understanding, interpretation, perception, meaning, relationship | **5** | perception* OR perceive OR perspective* OR understanding OR meaning OR experienc* OR belie* OR attitude* OR knowledge OR misconce* OR misunderstanding* OR expect* OR concept* OR view* OR opinion* OR value* OR relationship OR interpret* |
| **RESEARCH TYPE** | Qualitative research |  |  |
|  |  |  | **1 AND 2 AND 3 AND 4 AND 5** |

Table S2: Results of quality assessment (critical appraisal skills programme)

| **CASP Questions [4]** | **1.** Was there a clear statement of the aims of the research? | **2.** Is a qualitative methodology appropriate? | **3.** Was the research design appropriate to address the aims of the research? | **4.** Was the recruitment strategy appropriate for the aims of the research? | **5.** Was the data collected in a way that addressed the research issue? | **6.** Has the relationship between researcher and participants been adequately considered? | **7.** Have ethical issues been taken into consideration? | **8.** Was the data analysis sufficiently rigorous? | **9.** Is there a clear statement of findings? | **10.** How valuable is the research? |
| --- | --- | --- | --- | --- | --- | --- | --- | --- | --- | --- |
| Antin & Hunt, 2012 [5] | Y | Y | Y | Y | Y | N | Y | Y | Y | HIGH |
| Banna et al., 2016 [6] | Y | Y | ? | Y | Y | N | Y | Y | Y | MODERATE |
| Beagan & Chapman, 2012 [7] | Y | Y | ? | Y | Y | N | Y | Y | Y | HIGH |
| Brennan et al., 2020 [8] | Y | Y | Y | Y | Y | N | Y | Y | Y | HIGH |
| Caperchione et al., 2012 [9] | Y | Y | Y | Y | Y | N | Y | Y | Y | MODERATE |
| Delaney & McCarthy, 2014 [10] | Y | Y | Y | Y | Y | N | Y | Y | Y | HIGH |
| Dong et al., 2022 [11] | Y | Y | Y | Y | Y | N | Y | Y | Y | MODERATE |
| Fergus et al., 2021 [12] | Y | Y | Y | Y | Y | N | Y | Y | Y | MODERATE |
| Kombanda et al., 2022 [13] | Y | Y | Y | Y | Y | Y | Y | Y | Y | MODERATE |
| Koteyko, 2010 [14] | ? | Y | Y | Y | Y | Y | N | N | Y | LOW |
| Landry et al., 2018 [15] | Y | Y | Y | Y | Y | Y | Y | Y | Y | HIGH |
| Lee et al., 2023 [16] | Y | Y | Y | ? | Y | Y | Y | Y | Y | HIGH |
| Lucan et al., 2012 [17] | Y | Y | ? | Y | Y | ? | ? | Y | Y | MODERATE |
| McKenzie & Watts, 2021 [18] | Y | Y | Y | Y | Y | N | Y | Y | Y | HIGH |
| Mete et al., 2019 [19] | Y | Y | Y | Y | Y | N | Y | Y | Y | MODERATE |
| Niva, 2007 [20] | Y | Y | ? | Y | Y | N | N | Y | Y | LOW |
| Pettigrew et al., 2012 [21] | Y | Y | Y | Y | Y | N | Y | Y | Y | HIGH |
| Ristovski-Slijepcevic et al., 2008 [22] | Y | Y | ? | ? | Y | ? | Y | Y | Y | HIGH |
| Schoenberg et al., 2012 [23] | Y | Y | Y | Y | Y | N | ? | ? | Y | MODERATE |
| Sellaeg & Chapman, 2008 [24] | Y | Y | Y | Y | Y | Y | Y | Y | Y | MODERATE |
| Sogari et al., 2018 [25] | Y | Y | Y | Y | Y | Y | Y | Y | Y | HIGH |
| Stephens et al., 2018 [26] | Y | Y | ? | Y | Y | Y | Y | Y | Y | MODERATE |
| Winham et al., 2020 [27] | Y | Y | Y | Y | Y | Y | Y | Y | Y | HIGH |
| Wood et al., 2010 [28] | Y | Y | Y | Y | Y | Y | Y | Y | Y | HIGH |

*Y = Yes, N = No, ? = Can’t tell*

Table S3: Supporting participant quotations from primary studies by subthemes

| **(Sub)Theme** | **Supporting quotes** |
| --- | --- |
| **Theme 1: Constructions of healthy and unhealthy eating** | |
| Construction of healthy eating | A healthy diet *consists of lots of vegetables, but is above all varied—in a week’s period there’s quite a lot of various stuff included— and as little as possible salt and sugar and [y] that you eat when you’re hungry* [20]  *To me it’s something that’s in its most, you know, food that’s in its most natural state, like I understand that would be the most healthiest.* [19]  *… The process in which it’s cooked, depends if we’re having some grilled veggies or a stir fry with veggies, that’s healthy than a fried schnitzel.* [13]  *A healthy meal would be stir-fried vegetables with minimal added fat and a really good combination of different vegetables, different colours of veggies and types … with lean meat or lean fish … a chicken breast, cooked with minimal added fat and with rice … using brown rice instead of white rice … would be a healthy and a well-balanced meal.* [13]  […] *fruit salad, also, I like when there's many fruits together. So I think it's good and healthy at the same time. So there's no compromise.* [15] |
| Construction of unhealthy eating | *When I am trying to eat healthy I avoid all fast food, soda, or sugary drinks, no candy, or unnecessary ‘empty’ carbs*. [6]  *Processed foods like white pastas, white rice are not as healthy as whole grains … Processed meats, like salamis and sausages are not as healthy as other cuts of meat or no meat at all.* [13]  *Unhealthy foods … I think of chemicals or things that we read on the back of packages that we don’t really know … unusual scientific names … we should limit those sorts of foods*. [13] |
| Concepts of healthy eating | *Having a balanced diet in terms of you know fruit, veggies, fats, proteins, carbohydrates, that’s balance.* [19]  *It doesn’t make that much difference what you eat as long as you eat in moderation, that’s the basic thing.* [20] |
| **Theme 2: Considerations on dietary recommendations and healthy eating messages** | |
| Knowledge and awareness of healthy eating messages | *Eating 5 a day… Everybody’s aware of these things. Um … it’s in your consciousness now. It’s not even a food rule it’s just … it’s in you. You do have to think about it because you … you’re still ticking off one of your 5 a day because you’ve heard it so often, that’s what you do.* [18]  *I know what’s classed as healthy and unhealthy food, but there’s a lot of things, like margarines and butters and things like that, … low in saturates, polyunsaturates. What’s that? It’s just a word unless you know what they actually mean and what it can cause* [28]  *Well I drink a lot of soda, so probably not so much soda. And better food choices … I don’t necessarily know* [what healthier food choices are] [11]  *I classify food on how healthy it is depending on how much simple sugars are in it … I would consider pasta not healthy because it’s high GI, whereas something that was more meat-based with complex carbs rather than simple carbs as healthy* [13] |
| Attitudes and opinions towards healthy eating messages | In response to being shown a copy of the Australian Government’s Guide to Healthy Eating:  *When I look at this image, based off what I see before, I just think, "urgh", in resentment, as I have seen this so many times and I think the standards set out are just outrageous for a 'healthy lifestyle'. It's so hard to incorporate these foods into your daily life - and it's not a necessity, like, you're not going to be an obese person with heart disease if you don't follow the rules of the diet.* [8]  *One day they say this is good, but then the next day they say it is bad, very confusing*. [9]  *Canada’s Food Guide, which I have a real issue with that because of the way that it was put together. The beef industry and the dairy industry spent, you know, millions of dollars lobbying in Ottaway that’s incredibly biasing…those industries have been very successful at kind of still staying on that national agenda in that way* [22] |
| **Theme 3: Meanings attached to food and eating** | |
| Significance of healthy eating in daily life | *Because I do know what, I believe I know what healthy eating looks like, I just sometimes find it hard to translate it hard into practice and as, as you know that can be influenced by many things.* [19]  *I always struggle in eating healthy since I'm incredibly busy.* [8]  *It’s kind of in between because they* [neighborhood corner store] *do have fruits and vegetables, but they’re just expensive, and it’s cheaper to buy a bag of dollar potato chips than it is to spend like 3 bucks on 2 apples, you know.* [6]  *I just think it’s a common theme about people my age, and my circle of people are always aware of our weight and know what we should be eating, but still have a taste for the not so healthy stuff.* [26] |
| Eating as a pleasurable experience | *So it’s the taste, it’s the pleasure, it’s enjoyment that makes me buy whatever I buy, not the fact that it might or might not be good for me* [14]  *The right foods are the things that don’t taste good—fruits, vegetables, non fatty things. Things that you prepare in the home more than takeaways.* [21]  *I went and I got a churro [Mexican pastry]. I like churros, because they make me feel good, and they taste good. I don’t know, I was in a mood to have one. And I like it, because when you go there, they are nice and hot, and fresh. Usually, I would get this at the carnivals, and it kind of reminds me of when I’m at the carnival, and when I’m at a theme park, I always get one of those.* [5] |
| Food in stressful situations and as a means to provide comfort | *I work too much. I don’t take the down time to exercise. I like to snack a lot. I use food to regulate my mood.* [25]  *I went on an alkalising diet last year, and it was very strict. I lost way too much weight really quickly. It was too strict. However, you find, food is company, and when you cut it out, you have no comfort anymore. So, all these emotions come out ... I think that’s why things like chocolate and cakes and all the yummy things are quite comforting. I know through years of sort of struggling with weight, that eating is to push the emotions down. If you remove all of that, the emotions are coming out.* [16] |
| Negative feelings around food consumption | *So much of the way we look at food as pleasurable is about “indulgences” and “guilty pleasure” culture - “cheat days” or “i’m going to have to run this off later”. they all come with either guilt or shame or the idea of punishment after a satisfactory meal, as if food feeling good cant coexist with food being healthy. focusing on the feeling of having done something right rather than something wrong is really important.* [8]  *I’m addicted to bacon lately. I smell bacon and want it. I think that’s because there’s nothing really for people to do. But it’s like bloatiness and you know, drab and you feel depressed anyway if you eat the wrong things. Yeah an iffy tummy. I do regret it afterwards.* [28]  *There would be times where I would hit like an emotional low, and my way of coping with that was going and buying a block of chocolate and just eating it. Like the whole thing. However, then I feel guilt and shame for eating more than I should, and this makes me feel worse than I was feeling before.* [16] |
| Moral considerations in dietary behaviors | *So, you* [want to] *have a certain amount of the good stuff and only a small amount of the bad stuff.* [19]    *We try really hard to be good but then we’ll buy cakes and things so like cancel it out. Because we’re really good most of the time we can be quite naughty some of the time.* [14] |
| Health and well-being considerations in dietary behaviors | *I have a few family members that died … from high blood pressure, had a stroke, or diabetes or something you know. I feel like a big part of that is their diet. Like, I see it happening slowly. Like, if you eat too much of one thing, it’s not good for you if you don’t balance it out* […] [5]  *If you don’t eat the right types of food, then your brain’s not getting the right bits and pieces for it to work properly. That stuff, water, if I don’t get enough I get a headache.* [21]  *A healthy diet not only refers to eating well but also takes into consideration of exercising and getting adequate sleep. Those that get more exercise can allow more room to eat more and to then get a variety of food necessary for a healthy lifestyle.* [6]  *… Fruit is healthy because it’s natural … grows on the trees … natural, whereas the chocolate and the chips … very processed and don’t make me feel very good after I eat. I feel good when I eat healthy things and the unhealthy foods, I feel a bit sluggish or tired.* [13]  *It’s very individualistic. Everybody’s body processes are different. Some people react to one food in one way, and other people react to that same food in a different way. So, what is considered to be healthy, and what’s considered to be unhealthy may not be the same for every single person*. [16] |
| Social significance of healthy eating | *When you come from a loving home, then you see that in food. I honestly believe that there is love in the food that you serve, and that’s how I grew up.* [7]  *I think the big thing that changed for me was when I came here at Cornell, I saw other people and their eating habits, and some of them were eating lean or eating healthier, and I tried to pick up on some of those too.* [25]  […] *eating is definitely a social activity, so I love to cook but I'd also love to be able to share it with others*. [15]  *Things have changed. It might just be a reflection of my own friends, but I think a lot of guys I know cook more and want to eat a greater range of foods. I think there is a change where guys are picking up more responsibility at home.* [26]  *I think it all goes back to what you learned as a child and changing and starting your children off the right way*. [23] |
| Eating as a means to construct cultural identity | *Yes, there is [a Black way of eating]. It’s not healthy It’s a lot of deep-fried and fried foods and stuff like that … I just want to incorporate a more healthier way…* [The Black way of eating is important] *because food like cornbread and stuff like that and it seems like Black people are always—at any function they always have food … I don’t think it’s going to affect the culture if you stop—you know, we start eating a lot more healthier. I think it would prolong our lives. We have a high rate of heart disease.* [22]  *Even if we couldn’t afford it, we’d still eat fried chicken. And we’d still eat a lot of fried foods*. [27]  *I like hot meals, I like a lot of soul food, it reflects who I am and I’m not changing that for nobody … It’s very important for me to eat things that reflect who I am, my culture. I think it is very important for us to teach our kids that. This is what it’s called, this is where this comes from.* [7]  *I don’t believe in being skinny, a good wind storm and you blow away. You need to stay grounded, so when the wind comes up you can still hold yourself down. You know what I’m saying? I wouldn’t want my kids to be running around right thin and stuff.* [7] |

Table S4: Participants’ recollection of specific dietary recommendations

| **Content of dietary recommendations** |
| --- |
| Principles such as balance [9, 15, 20, 29], moderation [9, 20, 29], and variety [15, 20, 29] |
| Incorporation of all four food groups [7, 22] |
| Eat (more) fruits and vegetables [6, 7, 9, 11, 18, 20-22, 27, 28], including five a day [18, 21, 28] |
| Eat (plenty of) foods high in fiber [6, 9, 21, 22] |
| Less meat [7, 11] or moderate amounts of meat [21] |
| Eat moderate amounts of fish [21] |
| Avoid processed foods [9] and fast foods [11] |
| Limit alcohol intake [9] |
| Avoiding fat [7, 14, 18, 21, 22, 27], sugar/carbohydrates [11, 14, 18], and reducing salt intake [7, 18] |
| Fewer sweets [11] |

## References

1. World Bank. "World Bank Country and Lending Groups". <https://datahelpdesk.worldbank.org/knowledgebase/articles/906519-world-bank-country-and-lending-groups>. Accessed September 2, 2021.

2. OECD. "Our global outreach: Member countries". <https://www.oecd.org/about/members-and-partners/>. Accessed September 2, 2021.

3. Page MJ, McKenzie JE, Bossuyt PM, et al. The PRISMA 2020 statement: An updated guideline for reporting systematic reviews. *J Clin Epidemiol*. 2021;134:178-89.

4. Long HA, French DP, Brooks JM. Optimising the value of the critical appraisal skills programme (CASP) tool for quality appraisal in qualitative evidence synthesis. *Res Methods in Med Health Sci*. 2020;1(1):31-42.

5. Antin TMJ, Hunt G. Food choice as a multidimensional experience. A qualitative study with young African American women. *Appetite*. 2012;58(3):856-63.

6. Banna JC, Gilliland B, Keefe M, Zheng D. Cross-cultural comparison of perspectives on healthy eating among Chinese and American undergraduate students. *BMC Public Health*. 2016;16(1):1015.

7. Beagan BL, Chapman GE. Meanings of food, eating and health among African Nova Scotians: 'certain things aren't meant for Black folk'. *Ethn Health*. 2012;17(5):513-29.

8. Brennan L, Klassen K, Weng E, et al. A social marketing perspective of young adults' concepts of eating for health: is it a question of morality? *Int J Behav Nutr Phys Act*. 2020;17(1):44.

9. Caperchione CM, Vandelanotte C, Kolt GS, et al. What a Man Wants: Understanding the Challenges and Motivations to Physical Activity Participation and Healthy Eating in Middle-Aged Australian Men. *Am J Men's Health*. 2012;6(6):453-61.

10. Delaney M, McCarthy MB. Saints, sinners and non-believers: the moral space of food. A qualitative exploration of beliefs and perspectives on healthy eating of Irish adults aged 50–70. *Appetite*. 2014;73:105-13.

11. Dong KR, Chen X, Stopka TJ, Must A, Beckwith CG, Tang AM. Food Access, Dietary Intake, and Nutrition Knowledge of Adults on Probation. *J Nutr Educ Behav*. 2022;54(6):510-20.

12. Fergus L, Roberts R, Holston D. Healthy Eating in Low-Income Rural Louisiana Parishes: Formative Research for Future Social Marketing Campaigns. *Int J Env Res Public Health*. 2021;18(9):4745.

13. Kombanda KT, Margerison C, Booth A, Worsley A. How young adults in Australia classify foods as healthy and unhealthy. *Appetite*. 2022;175:106060.

14. Koteyko N. Balancing the good, the bad and the better: A discursive perspective on probiotics and healthy eating. *Health:*. 2010;14(6):585-602.

15. Landry M, Lemieux S, Lapointe A, et al. Is eating pleasure compatible with healthy eating? A qualitative study on Quebecers' perceptions. *Appetite*. 2018;125:537-47.

16. Lee MF, Bradbury JF, Yoxall J, Sargeant S. "It’s about What You’ve Assigned to the Salad": Focus Group Discussions on the Relationship between Food and Mood. *Int J Env Res Public Health*. 2023;20(2):1476.

17. Lucan SC, Barg FK, Karasz A, Palmer CS, Long JA. Perceived influences on diet among urban, low-income African Americans. *Am J Health Behav*. 2012;36(5):700-10.

18. McKenzie JS, Watts D. Food ideals, food rules and the subjective construction of a healthy diet. *Food Foodways*. 2021;29(1):66-86.

19. Mete R, Shield A, Murray K, Bacon R, Kellett J. What is healthy eating? A qualitative exploration. *Public Health Nutr*. 2019;22(13):2408-18.

20. Niva M. 'All foods affect health': Understandings of functional foods and healthy eating among health-oriented Finns. *Appetite*. 2007;48(3):384-93.

21. Pettigrew S, Pescud M, Donovan RJ. Older people's diet-related beliefs and behaviours: Intervention implications. *Nutr Diet*. 2012;69(4):260-4.

22. Ristovski-Slijepcevic S, Chapman GE, Beagan BL. Engaging with healthy eating discourse(s): Ways of knowing about food and health in three ethnocultural groups in Canada. *Appetite*. 2008;50(1):167-78.

23. Schoenberg NE, Howell BM, Swanson M, Grosh C, Bardach S. Perspectives on Healthy Eating Among Appalachian Residents. *J Rural Health*. 2013;29(s1):s25-s34.

24. Sellaeg K, Chapman GE. Masculinity and food ideals of men who live alone. *Appetite*. 2008;51(1):120-8.

25. Sogari G, Velez-Argumedo C, Gómez M, Mora C. College Students and Eating Habits: A Study Using An Ecological Model for Healthy Behavior. *Nutrients*. 2018;10(12):1823.

26. Stephens LD, Crawford D, Thornton L, et al. A qualitative study of the drivers of socioeconomic inequalities in men’s eating behaviours. *BMC Public Health*. 2018;18(1).

27. Winham DM, Knoblauch ST, Heer MM, Thompson SV, Der Ananian C. African-American Views of Food Choices and Use of Traditional Foods. *Am J Health Behav*. 2020;44(6):848-63.

28. Wood F, Robling M, Prout H, Kinnersley P, Houston H, Butler C. A Question of Balance: A Qualitative Study of Mothers' Interpretations of Dietary Recommendations. *Ann Fam Med*. 2010;8(1):51-7.

29. Lucan SC, Barg FK, Karasz A, Palmer CS, Long JA. Concepts of Healthy Diet Among Urban, Low-Income, African Americans. *J Community Health*. 2012;37(4):754-62.
